# Setting of an endoscopic nasal reference point for surgical access to the anterior base through an anatomical study on cadavers^☆^

**DOI:** 10.1016/j.bjorl.2015.10.021

**Published:** 2016-05-06

**Authors:** Andressa Vinha Zanuncio, Paulo Fernando Tormin Borges Crosara, Helena Maria Gonçalves Becker, Celso Gonçalves Becker, Roberto Eustáquio dos Santos Guimarães

**Affiliations:** a Universidade Federal de São João del-Rei (UFSJ), Campus Centro-Oeste, Divinópolis, MG, Brazil; b Universidade Federal de Minas Gerais (UFMG), Faculdade de Medicina, Departamento de Oftalmologia e Otorrinolaringologia, Belo Horizonte, MG, Brazil

**Keywords:** Skull base, Endoscopic surgery, FESS, Paranasal sinuses, Facial sinuses, Base do crânio, Cirurgias endoscópicas, FESS, Seios paranasais, Seios da face

## Abstract

**Introduction:**

Diseases of paranasal sinuses, nasal cavity, and skull base can be treated by endonasal operations using a nasal rigid endoscope. When conducting this kind of surgery, anatomical references are critical for safety.

**Objective:**

To measure the distance from the posterior wall of the maxillary sinus to the skull base, according to socio-demographic characteristics, and to detail an anatomical reference point for paranasal sinus operations and for an access to the anterior skull base, comparing anatomical variations between right and left sides, gender, height, weight, age, and ethnicity in cadavers.

**Methods:**

Measures were taken from the 90° angle (the starting point where deflection of the skull base begins to form the anterior wall of the sphenoid, also known as Δ90°) to the upper, middle, and lower points of the posterior wall of the maxillary sinus. This study used 60 cadavers aged over 17 years, and evaluated these bodies with respect to age, height, BMI, weight, gender, and ethnicity, comparing measurements of right and left sides.

**Results:**

The measurements were >1.5 cm in all cadavers and did not vary with age, height, weight, gender, and ethnicity on their right and left sides. The lack of association between the measurement from Δ90° to the upper, middle, and lower posterior walls of the maxillary sinus (categorical or quantitative) is noteworthy, considering the characteristics studied.

**Conclusion:**

The methodology defined the nasal point of reference, considering an absence of variation in the cadavers’ characteristics.

## Resumo

**Introdução:**

Doenças dos seios paranasais, cavidades nasais e doenças da base do crânio podem ser tratadas com operação endonasal utilizando-se endoscópio rígido nasal. Referências anatômicas são importantes para a segurança durante a realização dessas operações.

**Objetivo:**

Medir a distância da parede posterior do seio maxilar à base anterior do crânio de acordo com características sócio-demográficas. Detalhar um ponto de referência anatômico para operações dos seios paranasais e acesso à base anterior do crânio comparando variações anatômicas entre os lados direito e esquerdo, gênero, altura, peso, idade e etnia em cadáveres.

**Método:**

Medidas do ângulo de 90° (ponto onde inicia a deflexão da base do crânio para formar a parede anterior do esfenoide, chamado de ângulo de 90° – Δ90°) aos pontos superior, médio e inferior da parede posterior do seio maxilar. Foram utilizados 60 cadáveres com idade acima de 17 anos, e avaliados com idade, altura, peso IMC, gênero e etnia, comparando-se as medidas dos lados direito e esquerdo.

**Resultados:**

As medidas foram maiores que 1,5 cm em todos os cadáveres e não variaram com a idade, altura, peso, gênero e etnia nos lados direito e esquerdo dos cadáveres. Destaca-se falta de associação entre a medida do Δ90° à parede posterior superior; média e inferior do maxilar (categórico ou quantitativo) com as características estudadas.

**Conclusão:**

A metodologia empregada definiu o ponto de referência nasal por não variar com as características dos cadáveres.

## Introduction

Endonasal surgery guided by a nasal rigid endoscope (called endoscopic sinus surgery) is used for the treatment of diseases of paranasal sinuses, nasal cavities, and skull base diseases. Thus, one must have a detailed knowledge of nasal anatomy; a CT scan of facial sinuses and a nasolaryngoscopy study are indispensable for this procedure. The anatomy of the sinuses varies individually.^1^, ^2^, ^3^

Functional endoscopic sinus surgery is used to treat chronic rhinosinusitis with or without nasal polyps, for resection of nasal and paranasal sinus tumors, in malformations of the nasal cavity such as choanal atresia, in various inflammatory and infectious diseases of nasal cavity and paranasal sinuses, and in skull base diseases, with less morbidity/complications. Skull base surgery and revision procedures with distorted anatomy are the procedures most in need of precise anatomical references.^4^, ^5^, ^6^

Knowledge of fixed measures (with slight variation in characteristics such as gender, ethnicity, age, weight, and height) such as the distance from the posterior wall of the maxillary sinus to Δ90° (starting point where the deflection of the skull base begins, to form the anterior wall of the sphenoid) in the anterior skull base, would provide greater safety to surgeons. With such knowledge, iatrogenic complications to the posterior sinuses could be minimized. These measures have not been described in the literature, and will be presented in this article.^7^, ^8^, ^9^

The aim of this study was to measure distances from three points of the right- and left-side posterior wall of the maxillary sinuses to the anterior skull base (Δ90°) and compare them with the sociodemographic characteristics of interest; set other benchmarks for endoscopic surgical access; compare the anatomical variations of the reference points measured in relation to gender, height, weight, age, and ethnicity on cadavers; and to detail a new anatomical reference point to perform surgeries of the paranasal sinuses and anterior skull base in a safer environment.

## Methods

This study was approved by the Research Ethics Committee of the institution under protocol No. 0591.0.203.000-8.

**Figure 1:**
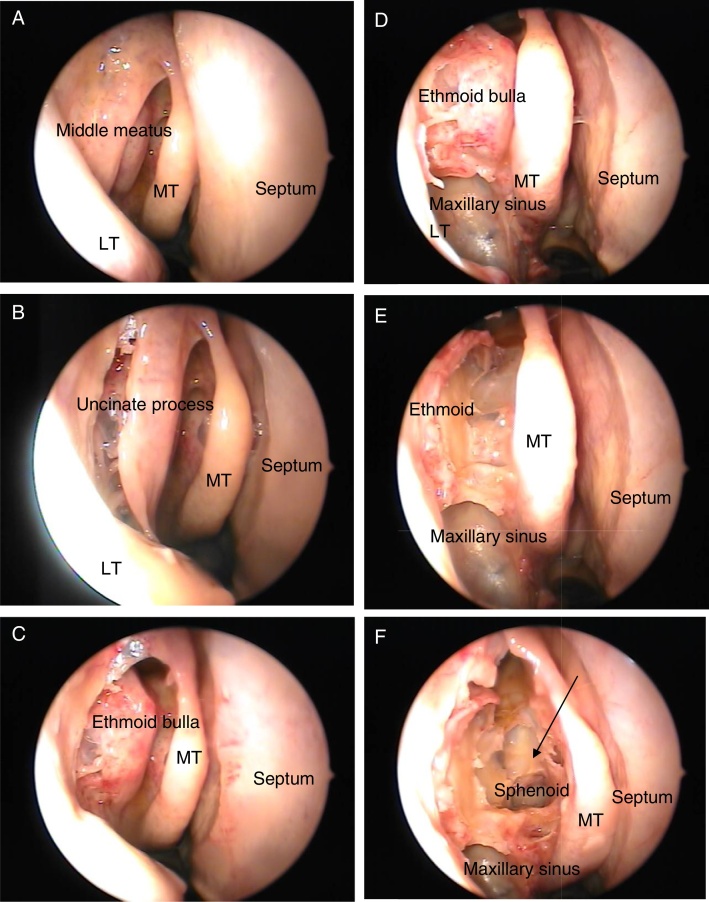
Right nasal cavity (MT, middle turbinate; LT, lower turbinate). (A) The beginning of dissection; (B) uncinate process; (C) ethmoid bulla; (D) maxillary sinus exposure; (E) ethmoid sinus exposure; (F) sphenoid sinus exposure. The arrow indicates the Δ90°.

The nasal cavities (right and left) were dissected in 60 cadavers, all aged over 17 years, of varying age, ethnicity, height, and gender. The medial wall of the maxillary sinus was opened, and anterior and posterior ethmoid sinuses and the sphenoid sinus were dissected to make it possible to identify the point where the deflection of the skull base begins, to form the anterior wall of the sphenoid, known Δ90° (Fig. 1), and to measure the distance from that point to the upper, middle, and lower points of the posterior wall of the maxillary sinuses.

The dissection was performed with microsurgical material through nasal endoscopy with zero-degree optics coupled with a camera, with DVD recording.

The framework for this study was established in the autopsy room with a video camera, a notebook, a nasal aspirator, a light source, surgical instruments, zero-degree optics, and fiber optic light cable. After the autopsy, the cadavers underwent paranasal sinus dissection, and at this moment the measures were taken.

**Figure 2:**
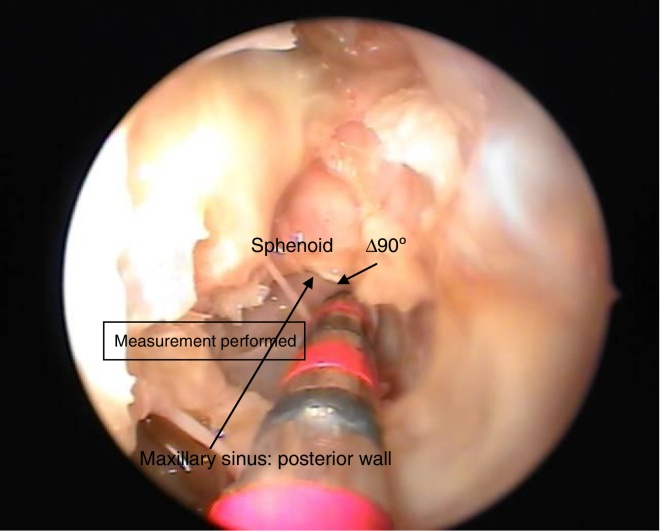
Performance of measures on the cadavers studied – Δ90° (the starting point where the deflection of the skull base begins to form the anterior wall of the sphenoid).

The following measurements were performed (Fig. 2): upper, middle, and lower parts of the posterior wall of the maxillary sinus to the angle of 90°, based on a previously stipulated protocol.

Covariates height, weight, age, body mass index (BMI), gender, and ethnicity were assessed in relation to the three measures studied.

The descriptive results presented in the Results section were obtained through the use of frequencies and percentages for the characteristics of the various categorical variables, and from the performance of central tendency measures (mean and median) and dispersion measures (standard deviation) for the quantitative variables.

The Pearson correlation coefficient (*r*) was used to compare the measurements and characteristics in quantitative forms (age, height, weight, and BMI).

Student's *t*-test was used for comparisons of measures in quantitative form with gender and ethnicity, since the usual assumptions of this test were met (normality – Shapiro–Wilk test and homoscedasticity – Levene).^10^

The three selected measures were dichotomized into two groups: ≥2 and <2, considering that, in surgical practice it was observed that generally the distances were ≥2.

## Results

### Descriptive analysis

There was a prevalence of male (55%) and white (78.3%) individuals. Mean age, height, weight, and BMI of the cadavers was 64 years; 1.70 m; 67.1 kg, and 22.5, respectively. There was a prevalence of cadavers aged between 48 and 88 years old, with height between 1.70 and 1.75 m, weight between 60 and 80 kg, and BMI between 18 and 26.

### Descriptive statistics between right and left sides

The cadavers had the following measures on their right side: a mean of 2.1 cm from Δ90° to the upper posterior wall of the maxillary sinus; a mean of 1.9 cm from Δ90° to the middle posterior wall of the maxillary sinus; and a mean of 1.7 cm from Δ90° to the lower posterior wall of the maxillary sinus. On the left side, these measures were 2.2 cm, 1.7 cm, and 1.6 cm, respectively (Table 1).

**Table 1:** Descriptive statistics of the features of the measurement of the distance from Δ90° to the superior (SPW), middle (MPW), and lower (LPW) posterior wall of maxillary sinuses of the studied cadavers on both (right and left) sides.

Features	*n*	Mean	SD	Min	1Q	Med	3Q	Max
*Right side*								
Δ90° (SPW)	60	2.1	0.3	1.5	2.0	2.0	2.5	3.0
Δ90° (MPW)	60	1.9	0.5	1.0	1.5	2.0	2.0	3.0
Δ90° (LPW)	60	1.7	0.5	0.5	1.5	1.5	2.0	2.5

*Left side*								
Δ90° (SPW)	60	2.2	0.4	1.5	2.0	2.0	2.5	3.0
Δ90° (MPW)	60	1.7	0.4	1.0	1.5	2.0	2.0	2.5
Δ90° (LPW)	60	1.6	0.4	0.5	1.5	1.5	2.0	2.5

*n*, number of observations; SD, standard deviation; Min, minimum; 1Q, 1st quartile; Med, median; 3Q, 3rd quartile; Max, maximum.

The measure from Δ90° to the upper posterior wall of the maxillary sinus was ≥2 cm on both sides in 91.7% of the studied cadavers. When measuring the difference (by side) between the distance from Δ90° to the middle posterior wall of the maxillary sinus and the distance from Δ90° to the lower posterior wall of the maxillary sinus, it was found that in 61.7% of the studied cadavers on the right side and in 51.7% on the left side, the distance from Δ90° to the middle posterior wall of the maxillary sinus was ≥2 cm. Furthermore, the distance from Δ90° to the lower posterior wall of the maxillary sinus was ≥2 cm in 46.7% and in 41.7% of right and left sides, respectively (Table 2).

**Table 2:** Categorized description of the measurement of the distance from Δ90° to the upper, middle, and lower posterior walls of the maxillary sinus of the studied cadavers on both (right and left) sides.

	Frequency			
	Right side		Left side	
Features	*n*	%	*n*	%
*Δ90 upper*				
<2	5	8.3	5	8.3
≥ 2	55	91.7	55	91.7

*Δ90 middle*				
<2	23	38.3	29	48.3
≥ 2	37	61.7	31	51.7

*Δ90 lower*				
<2	32	53.3	35	58.3
≥ 2	28	46.7	25	41.7

*n*, number of observations.

In ten (16.7%) of the 60 studied cadavers, the measure from Δ90° to the upper posterior wall of the maxillary sinus was <2 cm and >1.5 cm.

### Comparisons between sides

The measure from Δ90° to the middle posterior wall of the maxillary sinus showed a variation between sides, with a lower value on the left *versus* right side, with a difference of 0.2 (1.9–1.7) cm for this feature, with a statistically significant difference. No difference was observed in the other two measures (Table 3).

**Table 3:** Comparison of the measurement of the distance from Δ90° to the upper, middle, and lower posterior walls of the maxillary sinus of the studied cadavers by side (right and left).

	Side						
	Right			Left			
Features	Mean	SD	Med	Mean	SD	Med	*p*-Value
Δ90° upper	2.1	0.3	2.0	2.2	0.4	2.0	0.277^a^
Δ90° middle	1.9	0.5	2.0	1.7	0.4	2.0	**0.020**^a^
Δ90° lower	1.7	0.5	1.5	1.6	0.4	1.5	0.417^2^

SD, standard deviation; Med, median.

a Paired *t*-test.

### Comparisons of features with variable responses by side

#### Right and left sides – quantitative

The coefficient and *p*-values of Pearson's correlation (*r*) showed no association between measures from Δ90° to the upper, middle, and lower posterior wall of right and left maxillary sinuses and age, height, weight, BMI, gender, and ethnicity of the cadavers (*p* > 0.05) (Table 4).

**Table 4:** Summary of comparisons among the three evaluated measures and features of interest.

Variable responses	Features of interest (*p*-value)						
	Side	Age	Height	Weight	BMI	Gender	Ethnicity
From Δ90° to the upper posterior wall of the maxillary sinus (categorical or quantitative)	Right	0.814	0.681	0.217	0.081	0.482	0.987
	Left	0.762	0.225	0.976	0.415	1.00	0.304

From Δ90° to the middle posterior wall of the maxillary sinus (categorical or quantitative)	Right	0.268	0.643	0.746	0.916	0.585	0.802
	Left	0.480	0.342	0.194	0.347	0.517	0.501

From Δ90° to the lower posterior wall of the maxillary sinus (categorical or quantitative)	Right	0.068	0.295	0.967	0.502	0.317	0.800
	Left	0.389	0.338	0.313	0.551	0.916	0.083

#### Right and left sides – categorical

The measure from Δ90° to the upper, middle, and lower posterior wall of right and left maxillary sinuses was not associated with age, height, weight, BMI, race, and gender of the cadavers (*p* > 0.05).

## Discussion

This research was developed after an observation, during approximately ten years of nasal endoscopic surgeries, that the anatomical measures discussed were constant. Measures from Δ90° to the posterior wall of the maxillary sinus were similar, regardless of gender, ethnicity, age, weight, or height; but other measures varied with such features. Observations in surgical practice led the authors to perform these anatomical measurements on cadavers, seeking evidence in favor or against these observations, because the medical literature does not provide definitions on this topic. The corroboration of the regularity of the measures would allow the attainment of a more precise and constant anatomical reference, probably implying greater safety in the approach to posterior paranasal sinuses, chiefly by the fact that this area shows a great anatomic variation. In cases where there is a distortion of the anatomy, as in reoperations, this measure becomes even more useful.

Other measures taken in the nasal cavities of 60 cadavers have shown the influence of personal characteristics. However, there was no change in the distance from Δ90° to the upper posterior wall of the maxillary sinus, providing evidence in favor of what was already seen in this clinical practice. It was observed that 10% of the measures from Δ90° to the upper posterior wall of the maxillary sinus were <2 cm and >1.5 cm; thus, the fixed measure was changed to ≥1.5 cm.

In the measurement of the distance from Δ90° to the lower and middle posterior wall of the maxillary sinus, it was found that measures <1.5 cm were outliers. These measures have little accuracy, and are not suitable to be obtained during surgery because of their anatomical position in relation to Δ90°. Thus, the performance of such measures is not feasible during surgical procedures. Therefore, it was decided to use the 1.5-cm measure with respect to the upper point of the posterior wall of the maxillary sinus to Δ90°, thanks to its viability and also due to the absence of outlier values.

In surgical cases in which a maxillary sinus approach is not used, other anatomical structures should be used.

## Conclusion

The analysis of the data presented in this study allows for the conclusion that there is a fixed measurement between the upper posterior wall of the maxillary sinus and Δ90°. The value found was always greater than 1.5 cm, which can facilitate a safe opening of the posterior paranasal sinuses during nasal endoscopic surgeries with a maxillary sinus approach. The measurements of lower and middle points with respect to the posterior wall of the maxillary sinus should not be used in surgical practice, because of measurement difficulties due to their anatomical position.

Both categorically and quantitatively, there was no statistical association with respect to the difference between the measurements from Δ90° to the upper, middle, and lower posterior walls of the maxillary sinus in any of the evaluated characteristics. Thus, there was no impact from age, weight, height, ethnicity, or gender on these measures.

The definition of a fixed measure in paranasal sinus anatomy in an area where there is a greater chance of occurrence of iatrogenic error could impart a sense of greater safety to the surgeon and fewer complications in nasal endoscopic surgery.

## Conflicts of interest

The authors declare no conflicts of interest.
